# In Silico Analysis of Protein–Protein Interactions of Putative Endoplasmic Reticulum Metallopeptidase 1 in *Schizosaccharomyces pombe*

**DOI:** 10.3390/cimb46050280

**Published:** 2024-05-12

**Authors:** Dalia González-Esparragoza, Alan Carrasco-Carballo, Nora H. Rosas-Murrieta, Lourdes Millán-Pérez Peña, Felix Luna, Irma Herrera-Camacho

**Affiliations:** 1 Laboratorio de Bioquímica y Biología Molecular, Centro de Química del Instituto de Ciencias (ICUAP), Benemérita Universidad Autónoma de Puebla, Puebla 72570, Mexico; dalia.gonzalezesp@alumno.buap.mx (D.G.-E.); nora.rosas@correo.buap.mx (N.H.R.-M.); lourdes.millan@correo.buap.mx (L.M.-P.P.); 2 Laboratorio de Elucidación y Síntesis en Química Orgánica, Instituto de Ciencias de la Universidad Autónoma de Puebla (ICUAP), Benemérita Universidad Autónoma de Puebla, Puebla 72570, Mexico; 3 Consejo Nacional de Humanidades Ciencia y Tecnología, Instituto de Ciencias de la Universidad Autónoma de Puebla (ICUAP), Benemérita Universidad Autónoma de Puebla, Puebla 72570, Mexico; 4 Laboratorio de Neuroendocrinología, Facultad de Ciencias Químicas, Benemérita Universidad Autónoma de Puebla, Puebla 72570, Mexico; felix.luna@correo.buap.mx

**Keywords:** Ermp1, *S. pombe*, metalloprotease, protein–protein interaction, molecular docking, molecular dynamic

## Abstract

Ermp1 is a putative metalloprotease from *Schizosaccharomyces pombe* and a member of the Fxna peptidases. Although their function is unknown, orthologous proteins from rats and humans have been associated with the maturation of ovarian follicles and increased ER stress. This study focuses on proposing the first prediction of PPI by comparison of the interologues between humans and yeasts, as well as the molecular docking and dynamics of the M28 domain of Ermp1 with possible target proteins. As results, 45 proteins are proposed that could interact with the metalloprotease. Most of these proteins are related to the transport of Ca^2+^ and the metabolism of amino acids and proteins. Docking and molecular dynamics suggest that the M28 domain of Ermp1 could hydrolyze leucine and methionine residues of Amk2, Ypt5 and Pex12. These results could support future experimental investigations of other Fxna peptidases, such as human ERMP1.

## 1. Introduction

Endoplasmic Reticulum Metalloprotease 1 (ERMP1), also referred to as Fxna peptidase or Felix-ina, is a nine-transmembrane domain protease and a member of the M28 family of zinc metallopeptidases [1,2]. The gene was first discovered in the granulosa cells of rat ovarian follicles, where its expression is necessary for follicular organization, possibly to aid in the processing of protein precursors required for intraovarian cellular communication [2].

In vitro studies have suggested that overexpression of ERMP1 may contribute to increased endoplasmic reticulum (ER) stress by activating the Unfolded Protein Response (UPR) through GRP78-PERK-CHOP [1,3,4,5]. The role of the ERMP1 protein in cancer proliferation and progression through the PI3K/AKT/mTOR/β-catenin signaling pathways has been identified as a promising therapeutic strategy for treating various types of cancer [6,7]. Although there is evidence that ERMP1 could play an important role in these signaling pathways, the molecular mechanism remains unclear.

The use of Protein–Protein Interaction (PPI) networks has been instrumental in analyzing the molecular machinery of cells [8,9]. This approach is primarily employed to assign functional roles to potential proteins and to characterize multi-protein complexes and signaling pathways [10]. High-throughput technologies are available for the detection of PPIs, including yeast two-hybrid, immunoprecipitation, X-ray crystallography, and protein chips [11,12]. However, these techniques are limited to the analysis of reduced proteome coverage due to the time and cost required to perform experiments in the laboratory. Consequently, these limitations have prompted the development of computational tools for predicting large-scale PPIs, such as STRING, PEPPI, and deep neural networks [13,14,15]. Even so, the accuracy and reliability of these tools are highly dependent on the prior knowledge of the analyzed proteins. In contrast, there are databases that compile the interactions reported in publications based on experimental evidence, such as BIND, DIP, INTACT, MINT, and BioGRID [11,16,17,18,19,20].

In addition to the empirical discovery of PPIs, the comparative strategy of protein interactions conserved across species (interologues) has contributed to the functional annotation of uncharacterized proteins [21]. Although the prediction of human interologues inferred from model organisms is less frequent than expected, the high conservation of small groups of orthologues could be related to highly evolutionarily conserved cellular processes [9,21,22,23].

In particular, *Schizosaccharomyces pombe* is a fission yeast that shares many characteristics with eukaryotic cells, despite their evolutionary divergence of approximately 1144 million years [24]. Genetic conservation between *S. pombe* and humans has been confirmed by functional complementation of yeast mutants with human genes, showing high similarity between the two species [24,25,26,27]. Therefore, this model organism has been useful for the functional analysis of eukaryotic genes related to the cell cycle and the regulation of gene expression. In recent years, *S. pombe* has also been useful for the study of biochemical aspects of genetic products, such as the identification of domains related to catalytic activity and interactions with other proteins [28,29,30,31].

The orthologous gene *erm1* (SPCC1259.02c) in *S. pombe* encodes the protein Ermp1 (Putative Endoplasmic Reticulum Metallopeptidase 1), which has not yet been fully characterized. According to homology, it can be inferred that Ermp1 may be involved in proteolysis, as it contains the M28 domain commonly found in zinc metallopeptidases.

As the UPR signaling pathway is conserved in *S. pombe* [32,33,34], it is possible to study ER stress and its relationship with Ermp1. Since there is no experimental evidence of the cellular process in which it participates and its possible target proteins are not known, in the present study, an in silico prediction of the PPI using a comparison of the interologues between humans and yeasts, as well as the molecular docking and dynamics of the M28 domain of Ermp1 with fission yeast proteins.

## 2. Materials and Methods

### 2.1. Prediction of Protein–Protein Interaction

The bioinformatics assays were carried out in Laboratorio de Bioquímica y Biología Molecular and Laboratorio de Elucidación y Síntesis en Química Orgánica of the BUAP, Puebla, Mexico. The period of all online software was carried out in January to February 2024. The PPI network of human ERMP1 was obtained from the BioGRID 4.4 repository [20]. The queries were conducted on the platform between 2020 and 2023, taking into account the latest updates to the database. The DIOPT Ortholog Finder server 9.0, Boston, MA, USA [35] was used to search for orthologous proteins in *S. pombe* using the PPI network of human ERMP1 as a reference. DIOPT employs a suite of tools and algorithms, including Ensembl Compara v104, HomoloGene v68, Inparanoid v8, Isobase v2, OMA, orthoMCL v6.6, Phylome v4, RoundUp, and TreeFam v9, to predict orthologs across a range of species, including humans, mice, flies, worms, zebrafish, and yeasts [35]. These tools provide a simplified method for integrating, comparing, and accessing orthological predictions based on sequence homology, phylogenetic trees, and functional similarity. Additionally, DIOPT calculates a simple score indicating the number of tools supporting a given orthologous gene pair relationship. Moreover, Gene Ontology was used to assign the subcellular localization and related biological processes. A PPI network for the Ermp1 protein was constructed using orthologous proteins identified in *S. pombe*. The orthologous relationship score assigned by DIOPT was used as a distribution criterion. The PPI network was then edited using Cytoscape 3.10.

### 2.2. In Silico Prediction of Cleavage Sequences of Proteolytic Candidates

In order to identify potential proteolytic candidates for interaction analysis with the M28 proteolytic domain of Ermp1, we considered proteins with DIOPT scores greater than 9 [35] and located in the cytosolic compartment, as well as membrane proteins with cytosolic regions. The proteins selected were the first to be identified as true human homologs in *S. pombe* when the BioGRID repository was reviewed. To predict the cleavage sequences for the metalloprotease family, the amino acid sequences of each protein were analyzed using PROSPER, Victoria, Australia [36]. The results were selected according to a cleavage probability score greater than 0.8 [37] for the subsequent protein–protein docking analysis.

### 2.3. Three-Dimensional Modeling of the M28 Domain of Ermp1 and Proteolytic Candidates

The M28 domain of Ermp1 and the target proteins Ypt5, Pex12, Oca8, Fis1 and Pmc1 were modeled by homology in Phyre2 v2.0, London, UK [38]. The templates were selected based on the percentage of homology of the sequences, domain conservation, and cellular function. Additionally, validation reports of potential templates in the Protein Data Bank were reviewed to select those that met the acceptance criteria, such as the experimental method, structure resolution, and percentile scores (ranging from 0 to 100) of global validation metrics like R-value free, R-value work, R-value observed, and Ramachandran outliers. The models were validated using PDBeFold v2.59 [39] and the PROCHECK-SAVES v6.0 Structure Validation Server, Europe Union [40]. A Q-score of approximately 1 and an RMSD value < 1 Å were considered the main modeling validation parameters due to their association with the percentage of aligned residues and the quality of the alignment of the secondary structures with respect to the template [39,41,42]. Furthermore, it has been confirmed that the cleavage sequences predicted in PROSPER were present in the 3D models of the proteolytic candidates, which were subsequently utilized for the docking analysis. As the N-terminal segment of the protein could not be modeled by homology and was essential for the docking analysis, the 3D model of Amk2 was obtained from the AlphaFold Protein Structure Database 4.0, Europe Union [43]. The model was subjected to a validation analysis using PROCHECK-SAVES v6.0, with the confidence score pLDDT of AlphaFold (ranging from 0 to 100) serving as the criterion for assessment.

### 2.4. Analysis of the Sequence and Structure of the M28 Domain of Ermp1

A sequence alignment was conducted using NCBI-BLAST to determine the percentage of homology between the human ERMP1 (Q7Z2K6) and Ermp1 from the *S. pombe* (O94702) proteins. The M28 domain of both proteases was subjected to analysis, with supplementary information retrieved from the PhosphositePlus v6.7.3 database regarding the post-translational modifications of ERMP1 in humans [44]. Furthermore, a predictive analysis of the serine phosphorylation sites of both proteases was carried out on the NetPhos 3.1, Lyngby, Denmark [45], with results scoring > 0.9 considered to be measures of the prediction accuracy [46].

### 2.5. Optimizing the Conformational Stability of Proteins through Energy Minimization Techniques

The PDB files were prepared, and the energy was minimized at pH 7.4 using the Protein Preparation Wizard (PrepWizard) tool from Maestro 13.0, New York, NY, USA [47] before the molecular docking. The integrity of each of the structures was reviewed and adjusted. Hydrogen atoms were added for each protein and the conformations of the rotamers of the polar residues were verified. Furthermore, the protonation and tautomeric states of Asp, Glu, Arg, Lys, and His were adjusted to pH 7.4. Additionally, the orientation of the hydrogen bonds was adjusted using PROPKA at pH 7.4 and the proteins were minimized using the OPLS4 force field with an RMDS of 0.3 Å [48]. The models were edited and visualized using UCSF Chimera 1.17.1, San Francisco, CA, USA [49] and Maestro 13.0.

### 2.6. Protein–Protein Docking of Ermp1 and Proteolytic Candidates

The Blind Docking process was conducted on the ClusPro 2.0 Boston, MA, USA [50] and using BioLuminate 4.5 software New York, NY, USA [51] in accordance with previously established parameters for the protein–protein docking of zinc metalloproteases [52]. The M28 domain of Ermp1 was designated as the rigid receptor, while the target proteins were considered to be the ligands. The PDB files were imported into ClusPro 2.0, and protein–protein docking was initiated automatically with an RMSD of less than 10 Å between the receptor and the ligand. The most promising interaction models were selected from the balanced energy category, as this is the recommended approach for the analysis of enzyme complexes. The score calculated by the program using the PIPER algorithm was derived from the favored energy contributions (electrostatics-favored, hydro-phobic-favored, and van der Waals-electrostatics), and thus, it was not considered a measure of binding affinity. Because ClusPro 2.0 removes metal ions during the processing of PDB files, Zn^2+^ ions from the M28 domain of Ermp1 were added to the interaction complexes according to the model coordinates. In the BioLuminate 4.5 software, the protein–protein docking runs were conducted with a limit of 70,000 rotations for each ligand evaluated. The electrostatic potential of the Zn^2+^ atoms of the M28 domain of Ermp1 was assigned for each inter-action complex using PIPER and Prime. The TOP30 ranking of the results obtained from each program was analyzed to search for interactions between the catalytic cavity of Ermp1 and the cleavage sequences of the proteolytic candidates that were predicted in PROSPER. The molecular interactions were analyzed using DIMPLOT tool of the software LigPlus v.2.2, Europe Union [53].

### 2.7. Solvation of Interaction Models in WaterMap

The Ermp1 interaction complexes were hydrated in the WaterMap tool, New York, NY, USA [54]. A molecular dynamics simulation was carried out in the presence of the solvent and a thermodynamic analysis of the water in the protein binding site was also performed [52,55]. The WaterMap calculations were run with the default simulation parameters: TIP4P solvent model at 300 K, 1 atmospheric pressure, and 2 ns simulation time. Subsequently, a cluster analysis was conducted to identify the principal hydration sites through the partitioning of the solvent density distribution within the binding cavity [54,56].

### 2.8. Molecular Dynamics Simulation

Molecular dynamics studies were conducted in Desmond, New York, NY, USA [57,58] to assess the stability of the interaction complexes: Ermp1–Pex12, Ermp1–Amk2 and Ermp1–Ypt5. The simulation was conducted within an orthorhombic box, with the ligand–protein complex situated in the center at a distance of 10 Å. Molecules of water were utilized to solvate the box, with a concentration of 150 nM of sodium chloride employed to simulate physiological conditions. The energy system was minimized using the OPLS4 force field with 2000 interactions, with a convergence criterion of 1 kcal/mol/Å [59]. Finally, a 120 ns simulation was conducted at 300 K and 1 bar of pressure. The root means square deviation (RMSD) and root mean square fluctuation (RMSF) were reported.

## 3. Results

### 3.1. Prediction of Protein–Protein Interactions of Ermp1 in S. pombe

BioGRID is an open-access database resource that hosts gene and protein interactions from multiple species, including yeasts, worms, flies, and humans. All the content is selected from experimental evidence reported in scientific publications, making it the most comprehensive repository of its kind [20]. Approximately 1.93 million of the reported interactions can be used to build interaction networks that facilitate biomedical research, particularly in the context of human health and diseases [20]. The BioGRID database currently contains a register of an interactome consisting of 19,229 interactions between 3844 proteins encoded in the yeast genome. Furthermore, it has been observed that at least 1355 proteins in the *S. pombe* proteome correspond to human orthologs that are related to diseases such as diabetes, hypertension, and cancer [27,29].

For example, the molecular study of *S. pombe* has proven invaluable for the functional analysis of genes related to human diseases, such as *RAD1*, *RAD9*, and *HUS1*. These human genes play a crucial role in the checkpoint response to DNA damage, and their aberrant expression has been linked to various cancers, including prostate, breast, intestinal, thyroid, and gastric cancers. Notably, these genes exhibit high homology with *rad1*, *rad9*, and *hus1* in *S. pombe* [60,61].

**Figure 1:**
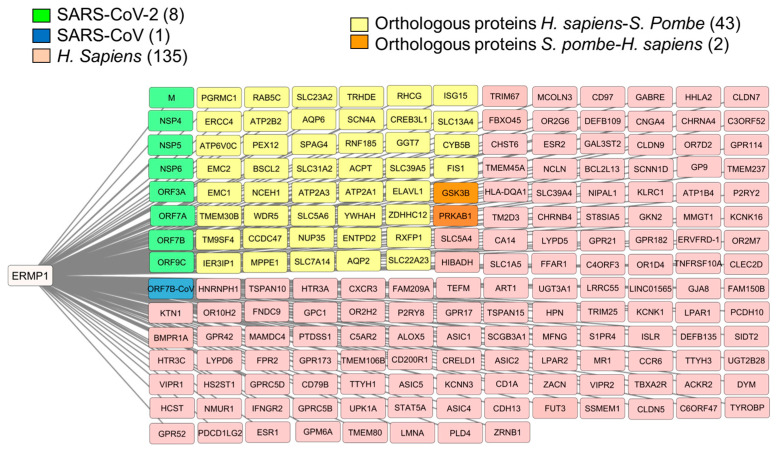
PPI network for human ERMP1. Information obtained from the BioGRID database. The network was edited using Cytoscape 3.10 software.

Between 2020 and 2023, BioGRID recorded 190 proteins with experimental evidence of interaction with ERMP1 in *H. sapiens* (Q7Z2K6), including 8 viral proteins from SARS-CoV-2 and 1 from SARS-CoV (Figure 1). Despite this, no evidence of interaction was observed with the major UPR pathway proteins, such as IRE1, PERK, ATF6 and GRP78. However, the network analysis revealed the presence of proteins that are related to the activation of the pathway in the ER in a non-canonical manner, such as TM9SF4 [62], IER3IP1 [63] and BSCL2 [64], although the exact mechanism of their involvement remains unclear.

According to BioGRID’s records, there are currently no known instances of Ermp1 PPI in *S. pombe* (O94702), but there are positive genetic interactions (GIs) with the *amk2* and *gsk3* genes. These interactions were identified in a synthetic genetic array (SGA) study of single and double mutants of genes related to the TORC1 (Rapamycin Complex) signaling pathway [65].

GIs can be classified as either negative (lethality) or positive (suppression). In the case of a negative GI, a double mutant exhibits a more severe phenotype than that observed in single mutants, indicating that the gene products function redundantly in parallel on a signaling pathway. In cases of positive GI, it appears that the phenotype of the double mutants is less severe than that observed in the single mutants. This may suggest that the gene products interact within the same signaling pathway [66,67,68].

Ermp1 is suggested to have a physical interaction with Amk2 and Gsk3, as there may be a synergy between the GI and PPI. As a result, these proteins were included in the PPI network. Furthermore, the corresponding orthologous proteins in humans, PRKAB1 and GSK3B, were added to the human ERMP1 interaction network (Figure 1, highlighted in orange). PRKAB1 serves as a subunit of the heterotrimeric AMPK complex, which acts as a scaffold for the assembly of the PRKAA1 and PRKAG1 subunits. The AMPK complex plays a role as a negative regulator of the UPR pathway [69]. Additionally, GSK3B, a kinase, has been associated with an increased apoptosis response during ER stress, although its influence on the signaling pathway is not yet fully understood [70].

**Figure 2:**
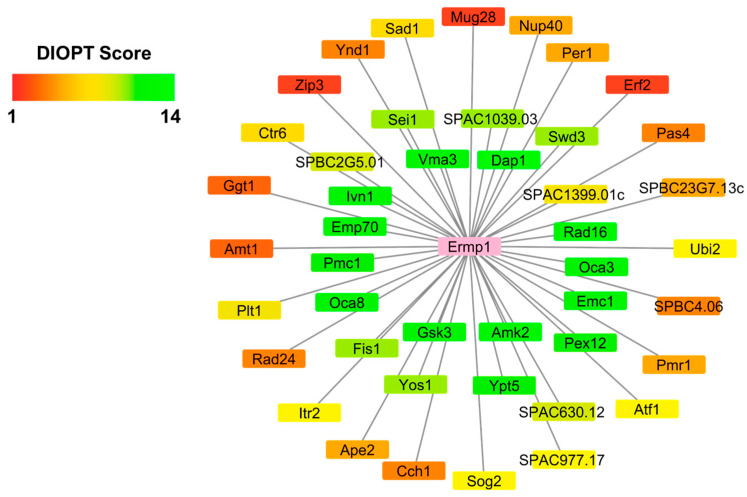
Prediction of a PPI network for Ermp1 in *S. pombe*. The network was edited using Cytoscape 3.10 software.

Analysis in DIOPT identified 45 human orthologous proteins in *S. pombe* (Figure 1, highlighted in yellow and orange, Table S1). The proteins were distributed in an interaction network based on the score assigned by DIOPT (Figure 2). Proteins with scores greater than 9 (Figure 2, highlighted in green, and Table S1) are considered true homologs, with conserved topological characteristics and cellular functions between fission yeast and humans.

**Figure 3:**
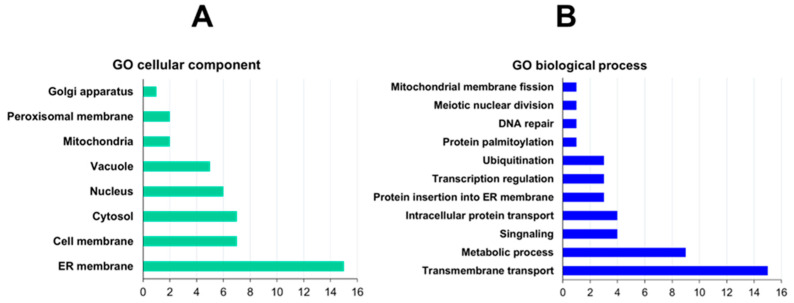
Clustering of Ermp1-interacting proteins in *S. pombe*: (**A**) Go cellular component and (**B**) biological process annotation.

The proteins were classified based on their subcellular localization and biological processes using the GO categories. A total of 29 proteins were found to be abundantly located in the ER membrane, cell membrane, and cytosol, while the remaining proteins were categorized in other cellular compartments such as the nucleus, vacuole, mitochondria, peroxisome, and Golgi apparatus (Figure 3A, Table S1). Fifteen proteins have been classified under transmembrane transport based on the biological process. The primary function of these proteins is to transport ions, including but not limited to Ca^2+^, Cu^2+^, Zn^2+^, and H^+^. Additional details can be found in Figure 3B and Table S1.

Mammalian cells rely on the endoplasmic reticulum (ER) as the primary organelle for Ca^2+^ storage due to the high oxidant potential required for the activity of numerous enzymes responsible for folding, post-translational processing, and protein trafficking [71]. Similarly, in *S. pombe*, calcium-dependent ATPases such as Pmr1 and Pmc1 are re-sponsible for regulating Ca^2+^ homeostasis in both the ER and vacuole [72]. Both transporters were identified in the *S. pombe* PPI network, which suggests a relationship between Ermp1 and Ca^2+^ homeostasis. On the other hand, 9 proteins associated with lipid and peptide metabolism were identified (Figure 3B). As previously noted, the ER plays a crucial role in various cellular processes, such as protein synthesis, Ca^2+^ storage, detoxification of chemical compounds, lipid synthesis, and lipid membrane assembly [73]. It is possible that the interaction of Ermp1 with metabolic proteins is linked to the maintenance of lipid and protein homeostasis within the organelle.

Considering that Ermp1 is a transmembrane protein, it is likely to interact with proteins involved in protein insertion into the ER membrane and vesicular trafficking. Moreover, Ermp1 could be regulated at the mRNA and protein levels through transcriptional regulation, ubiquitination, and palmitoylation mechanisms (Figure 3B). The proteins that were classified in a reduced proportion in the biological processes, such as DNA repair, nuclear division, and mitochondrial fission, could potentially interact with Ermp1 in the ER before being directed to different cellular compartments.

### 3.2. Comparing the M28 Domain of Ermp1 between Humans and S. pombe

Fxna peptidases are classified as members of the M28 family of metalloproteases due to their conservation of the His-Xaa-Asp and Glu-Glu motifs [74,75]. This family includes representative members such as aminopeptidase Y from *S. cerevisiae*, aminopeptidase S from *Streptomyces griseus*, aminopeptidase IAP from *E. coli*, and aminopeptidase Ap1 from *Vibrio proteolyticus*. The metalloproteases in question are characterized by the presence of two Zn^2+^ ions in their active center. These ions are tetrahedrally coordinated with three amino acids (His, Asp, and Glu) and a water molecule. Additionally, an aspartate or glutamate is coordinated to both metal ions [74,76].

**Figure 4:**
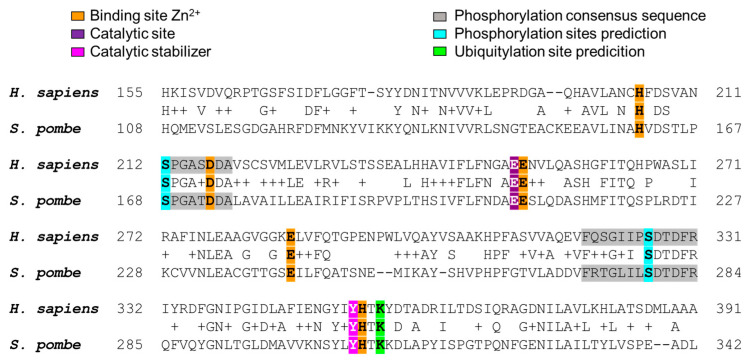
Sequence alignment of the M28 domain of Ermp1 in humans and *S. pombe*.

Since Fxna peptidases such as Ermp1 have not yet been assigned to a subfamily due to their putative status, their structural or enzymatic characterization is not referenced. Therefore, the M28 domain of human ERMP1 was compared to its orthologous pair in *S. pombe* based on a sequence alignment. Both proteins share 37% identity and retain the active center residues: His, Asp, and Glu, which bind to Zn^2+^. The residues Glu, His, and Tyr play important roles in the catalytic process (Figure 4, highlighted in orange, purple, and magenta, respectively).

According to experimental evidence provided by PhosphoSitePlus [77], it has been shown that the Ser326 residue of human ERMP1 is phosphorylated [78]. In light of this information, we decided to investigate whether Ermp1 from *S. pombe* could also contain this site. As shown in Figure 4, we aligned Ser326 with Ser279 of Ermp1 from *S. pombe*, which is located within a conserved consensus sequence (highlighted in cyan and gray, respectively). The sequences of the metalloproteases were analyzed using NetPhos 3.1 [45]. It was observed that one of the predicted potential phosphorylation sites matched the reference and alignment (Tables S2 and S3).

In addition, the analysis predicted the presence of a phosphorylation site for Ser212 of human ERMP1 and Ser168 of Ermp1 from *S. pombe* (Figure 4, highlighted in gray and cyan, respectively; Tables S2 and S3). The specific type of kinases that could target this type of metalloproteases is currently unknown. However, the PPI network proposed for Ermp1 in fission yeast includes the serine/threonine kinase Gsk3. It is worth noting that phosphorylation of Gsk3 substrates typically leads to their inactivation or degradation [79]. It is possible that under certain physiological conditions, Ermp1’s activity may be regulated by kinases, such as Gsk3, as part of the cellular metabolic and energetic modulation [80].

In the PPI network, Ubi2, a ubiquitin protein ligase, was identified. As a result, a search for ubiquitination consensus sequences for Ermp1 was also conducted. It is worth noting that, according to experimental reference in PhosphoSitePlus, the Lys356 residue of human ERMP1 can be ubiquitinated [81,82]. Furthermore, in the sequence analysis, the alignment of Lys356 with Lys309 of Ermp1 from *S. pombe* was observed (Figure 4, highlighted in green). It is possible that Ubi2 may be involved in the proteasomal degradation of Ermp1, as suggested by the predictions.

### 3.3. 3D Modeling of the M28 Domain of Ermp1 from S. pombe

To proceed with the analysis, it was necessary to obtain a three-dimensional structure of Ermp1. Unfortunately, there are no crystallographic structures of Fxna peptidases in the Protein Data Bank. Therefore, we opted for in silico modeling of the protein.

The AlphaFold artificial intelligence has been widely recognized for its ability to accurately predict complete 3D structures using a deep-learning neural network system [83]. However, the 3D model of Ermp1 (O94702) obtained from the AlphaFold database was found to be unsuitable for analyzing the M28 domain due to its conformation, which inhibits interaction with other proteins.

Due to the shortage of templates exhibiting high homology percentages, adequate modeling coverage, and validation metrics essential for generating the 3D model of the M28 domain of Ermp1 from *S. pombe*, the available templates were meticulously evaluated. The selection process prioritized templates that could yield validation parameters closely aligned with the ideal values of PDBeFold and PROCHECK. Consequently, the aminopeptidase Ap1 from *V. proteolyticus* (PDB ID: 1RTQ) was identified as the optimal template for homology modeling in Phyre2.

**Figure 5:**
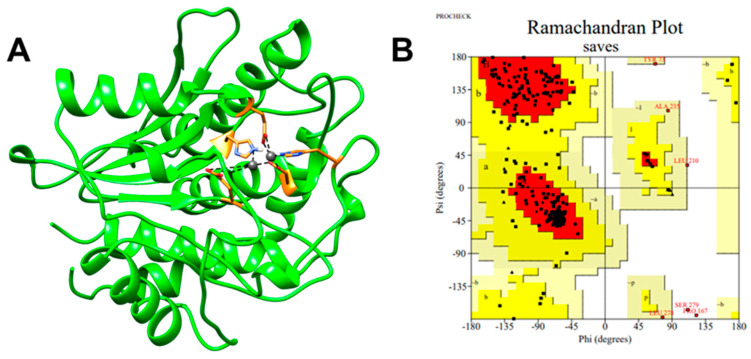
Three-dimensional structure of the M28 domain of Ermp1 from *S. pombe*. (**A**) Homology modeling in Phyre2. Binding site Zn^2+^ shown in orange. (**B**) Model validation using the PROCHECK-SAVES v6.0 server. Ramachandran plot shows 85.4% residues are in the most favored region. Editing of the model performed in UCSF Chimera 1.17.1.

The modeled sequence covered positions 39–339, with an identity percentage of 20% with respect to the template. A model was obtained with a Q-score of 0.76 and an RMSD of 0.72 Å. These parameters were observed to have a correlation with the percentage of aligned residues and the quality of the alignment of secondary structures with respect to the template. Figure 5A displays the 3D structure of the M28 domain of Ermp1, which shows the typical topology of metalloproteases. The enzyme is composed of β-sheets surrounded by α-helices, and its active center is represented by the Zn^2+^-binding residues located in the turns, highlighted in orange.

To ensure good quality, it is recommended that a model include more than 85–90% of its residues in the favored regions for each modeled secondary structure (β-sheets, α-helices, and loops) on a Ramachandran plot [84]. The model obtained from the M28 domain of Ermp1 in Phyre2 represents 85.4% of the residues in the favored regions in the Ramachandran plot (Figure 5B), which can be considered an acceptable model for the purposes of this study.

**Figure 6:**
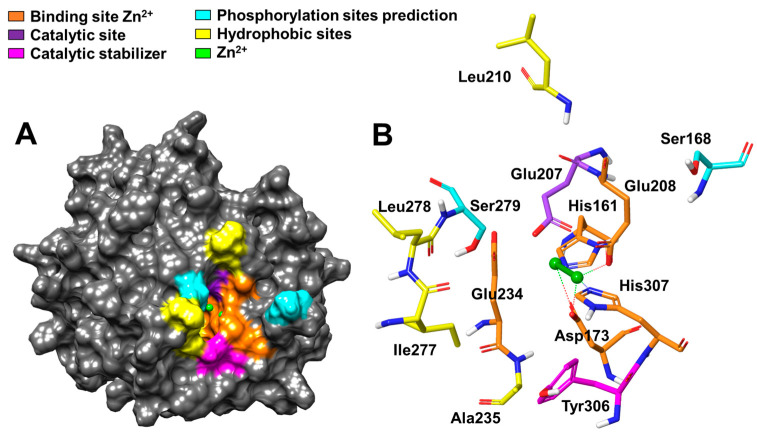
Representation of the catalytic cavity of the M28 domain of Ermp1 from *S. pombe*. (**A**) Surface model and (**B**) residues comprising the catalytic cavity. The gray dots represent the coordination bonds between residues with Zn^2+^. Editing of the model performed in Maestro 13.0.

Figure 6A displays the surface representation of the M28 domain of Ermp1, highlighting its globular conformation and the exposed active center available for interaction with the target proteins and peptides. The catalytic cavity of the protein, as shown in Figure 6A with orange, purple, and magenta highlights, is formed by several Zn^2+^-binding residues, including His161, Asp173, Glu208, Glu234, and His307, as well as Glu207 and Tyr306. This cavity restricts access to small peptides and secondary structures, such as protein loops. Figure 6B illustrates the amino acids that were identified in the catalytic cavity.

Based on the reference metalloprotease Ap1 from *V. proteolyticus* [85], Zn^2+^ is coordinated by Asp173, Glu208, and His307 to activate a molecule from H_2_O to OH^-^. Additionally, Glu207 plays a crucial role in accepting the proton, which is necessary to increase the polarity of the nucleophilic attack of Zn^2+^ on the peptide bond of the substrate. It is important to note that these findings are subject to further investigation and interpretation.

The additional Zn^2+^ ion coordinates with His161, Asp173, and Glu234 to lower the pKa of the H_2_O molecule, promoting greater specificity and stabilization of the catalytic reaction [86]. Furthermore, Tyr306 (highlighted in magenta in Figure 6A,B) plays a role in stabilizing the transition state of the substrate intermediate in the final catalysis step.

The hydrophobic amino acids corresponding to residues Leu210, Ala235, Ile277, and Leu278 (highlighted in yellow in Figure 6A,B) could potentially play a crucial role in maintaining the structural conformation of the catalytic cavity. Additionally, as shown in Figure 6A,B, there are potential phosphorylation sites located near the active center of Ermp1 (highlighted in cyan), specifically Ser168 and Ser279. It is possible that the proteolytic activity of the metalloprotease is regulated by some type of kinase.

### 3.4. Identification of Potential Proteolytic Cleavage Sequences and Generation of 3D Models

In the absence of methodological references for investigating protein–protein interactions involving zinc metalloproteases and target proteins, we conducted a prior study focusing on predicting consensus sequences with proteolytic susceptibility. This investigation utilized PROSPER in conjunction with molecular-docking tools such as ClusPro and BioLuminate, as previously reported [52]. The analysis aimed to identify consensus sequences of known proteins that can be cleaved by specific metalloproteases, for which there was experimental evidence of enzymatic interaction. The study showed that the tools used had a high predictive power. The results were similar to those of the published reports on the Metalloproteinase 8-Fibronectin 1 [87] and Metalloproteinase 12-Factor XII [88], which were used as validation controls.

Herein, the proteolytic analysis of selected proteins from the predicted Ermp1 PPI network was also performed. A comparative analysis was conducted between the cleavage segments of the proteins and their secondary structures, with a focus on the M28 domain of Ermp1. This domain has a narrow catalytic cavity, which affects the cleavage prediction. The consensus sequences were accepted for analysis if they were located in loops, as these structures have a greater probability of being cleaved. The PPI network proteins were selected for analysis based on the order of identification in the comparative analysis of human–yeast interologues since 2020. Table 1 displays the results of the proteolytic analysis of selected proteins from the Ermp1 PPI network, specifically the cleavage sequences predicted by PROSPER for the proteins Amk2, Ypt5, Pex12, Oca8, Fis1, and Pmc1. Metalloproteinase 9 was found to have the highest scores, with scores > 0.8. The majority of the proteolytic cleavages were found to be located at the N-amino terminus of the Met and Leu residues.

The predictions made in this study are consistent with the findings concerning other metalloproteases of the M28 family, such as the aminopeptidase Y from *S. cerevisiae* and the aminopeptidase Ap1 from *V. proteolyticus*. These proteases prefer cleaving the N-amino of Leu, but they are also capable of hydrolyzing the N-amino of Lys, Arg, Met, Val, and Ile [86,89]. Table 2 shows the templates that were used for the homology modeling of the proteolytic candidates in Phyre2, with the exception of Amk2.

From the list of templates returned by Phyre2, those that exhibited reliability per-centages approaching 100% were subjected to analysis. The reliability percentage calculated by Phyre2 considers the homology of the sequences between the template and the target; therefore, this percentage is distinct from the quality of the model. Moreover, due to the limited availability of templates with high levels of homology, models for some *S. pombe* proteins were selected based on their ability to achieve validation parameters that were as close as possible to the ideal values. All the models passed the validation and accuracy criteria, obtaining a Q-score > 0.7 and RMSD values < 1 Å. Additionally, in the Ramachandran plots, over 85% of the modeled residues of each protein were found in the favored regions for each secondary structure (please refer to Figure S1).

For molecular docking, the cleavable segments located in the loops were considered instead of the β-sheets and/or α-helices of the 3D models of the proteolytic candidates. This is because these regions are typically more accessible, flexible, and susceptible to proteolytic cleavage [95].

In contrast to other proteins, it is worth noting that the 3D model of Amk2 was obtained from AlphaFold (P78789). It is important to mention that the crystallographic structures of Amk2 from *S. pombe* are deposited in the Protein Data Bank (PDBs ID: 2OOX, 2OOY, 2QR1, 2QRC, 2QRD, and 2QRE); however, it should be noted that they do not include the cleavage sequence of interest. This is due to the fact that the sequence is situated in an area with a consistent structural transition between order and disorder, referred to as an intrinsically disordered region (IDR), which cannot be resolved through crystallography [96,97]. As a result, homology modeling was also deemed unsuitable for incorporating IDRs.

Although the Ramachandran plot of the AlphaFold model indicated that 81.1% of the residues were located in the most favored regions, this can be attributed to the presence of residues in the IDRs. As these regions are considered dynamic, they lack a defined secondary structure (see Figure S2). This is consistent with the pLDDT score < 50 calculated in AlphaFold for that region, which, according to the program, would correspond to an unstructured region.

In the past two decades, the existence and abundance of proteins with IDRs such as Amk2 have been demonstrated [96]. The discovery of these proteins challenged the classic concept that a well-defined structure is essential for a protein to perform its function. Currently, it is known that IDRs are functional, as they are considered dynamic structures that are usually at the connection points of interactions with other proteins, mainly in cell signaling pathways [97]. Consequently, AlphaFold has emerged as a valuable bioinformatics tool for the prediction and modeling of IDRs.

### 3.5. Protein–Protein Docking between the M28 Domain of Ermp1 and Proteolytic Candidates

To further evaluate the PROSPER proteolytic susceptibility prediction, a protein–protein rigid-docking analysis was conducted using the ClusPro 2.0 server and BioLuminate software. The M28 domain of Ermp1 was docked with each of the proteolytic candidates. Table 3 shows the blind-docking prediction results that were identified within the TOP30 of the ranking and the size of the clusters.

The scores obtained from ClusPro and BioLuminate were found to be similar, likely due to the use of the same prediction algorithm. However, it is important to note that these scores cannot be considered as measures of the binding affinity without the use of refinement methods for energy minimization. Additionally, while the docking programs did show interaction shifts of the cleavage sites (indicated by red arrows in Table 3), it is worth noting that these interactions were found within the cleavage consensus sequences predicted by PROSPER for each proteolytic candidate.

**Figure 7:**
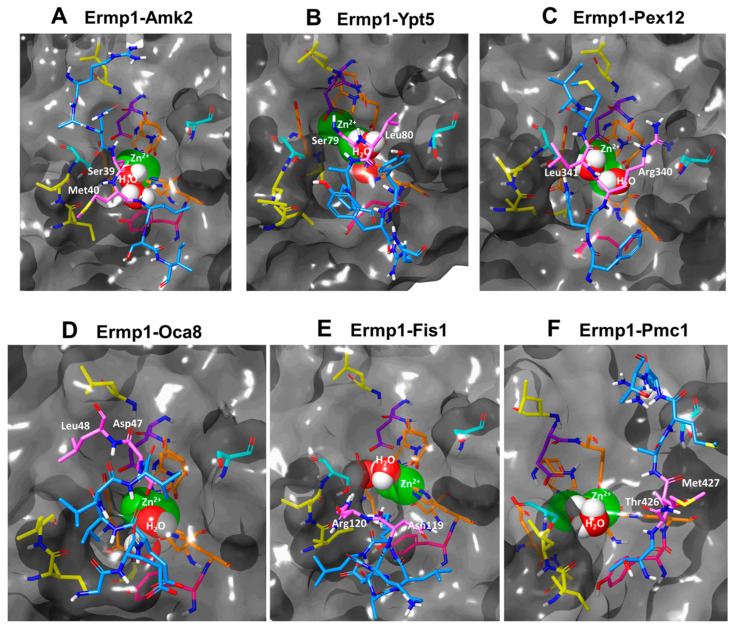
Molecular docking of the M28 domain of Ermp1 with protein targets (**A**–**F**). Highlighted are the binding sites for Zn^2+^ in orange, the catalytic site in purple, and the catalytic stabilizer in magenta. Additionally, the predicted phosphorylation sites are indicated in cyan. The hydrophobic areas are indicated by the yellow sites, and the ligand cleavage site is represented by the blue segment. The cleavage points are indicated by the pink residues. Solvation was performed using the WaterMap tool, and the models were edited in Maestro 13.0.

Figure 7A–F and Figure S3 display the cleavage sequences of the evaluated proteins. These sequences were located in flexible secondary structures, with the exception of Oca8 and Fis1, which were located in the short chains of the α-helices. It is worth noting that although proteolysis in α-helices is not common, the enzyme–substrate interaction may still occur because secondary structures in the solution are typically in a dynamic equilibrium that fluctuates between the α-helix and the loops [98,99].

The interaction complexes were identified near the N-amino terminus of the Leu, Met, and Arg residues of the target proteins (Figure 7A–F, Figure S3 and Figure S4). It appears that Ermp1 may prefer these amino acids, which are similar to those found in the Ap1 aminopeptidase of *V. proteolyticus* with respect to proteolysis.

During the simulation of the models in WaterMap, it was noted that hydration appeared to facilitate the interaction of H_2_O molecules with Zn^2+^ in the catalytic cavity of the M28 domain of Ermp1 (refer to Figure 7A–F).

In the hydration and water-exchange mechanism of Zn^2+^, the bond distance between Zn^2+^ and the oxygen of H_2_O ranges between 2.0 and 2.1 Å in the first coordination sphere of the metal, 3.6 Å in the second coordination sphere, and between 2.7 and 3.0 Å in the transition states between both spheres [100].

The distances between Zn^2+^ and water oxygen were measured in each of the docking models (see Figure S5). Variations in the length of the bonds were observed that ranged between 2.46 and 3.51 Å; therefore, the interactions would be found between the first and second coordination spheres of Zn^2+^. These results are consistent with other computational chemistry studies, where ligand exchange and catalytic mechanisms are favored in the transition states of both coordination spheres [101,102,103].

**Figure 8:**
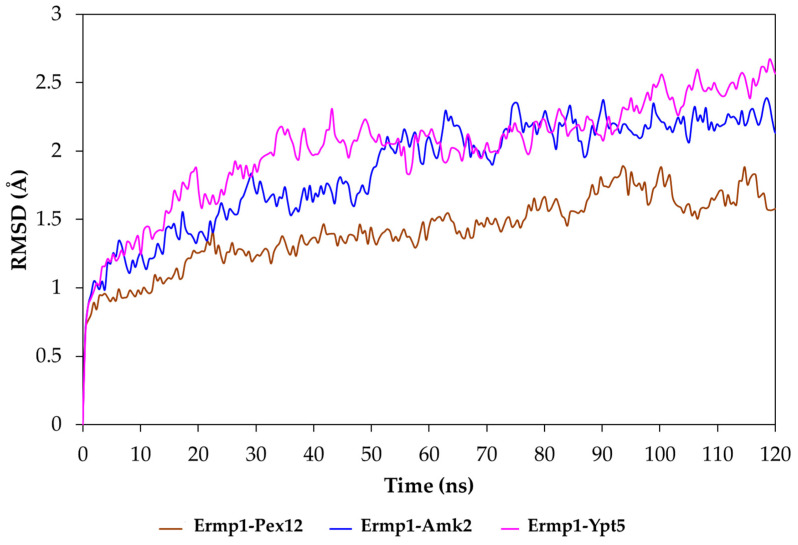
Molecular dynamics simulation. Variation in the root mean square deviation (RMSD) of the Ermp1–Pex12, Ermp1–Amk2 and Ermp1–Ypt5 complexes. Each simulation was 120 ns long in the Desmond Molecular Dynamics System to assess the stability of the protein–protein interactions.

The results of the protein–protein docking procedure indicated that three systems were worthy of further investigation through molecular dynamics studies. The Ermp1–Amk2, Ermp1–ypt5, and Ermp1–Pex12 systems were selected based on the observation of highly interactive behavior between the catalytic cavity of the M28 domain of Ermp1 and the cleavage sequences present in the target proteins. The root mean square deviation (RMSD) of each snapshot relative to the initial energy-minimized structure was calculated following alignment based on the Cα atoms for each trajectory. As depicted in Figure 8, the RMSD values for all the systems exhibited reasonable fluctuations, ranging from 0.8 to 2.6 Å throughout the simulations. It can be noted that the interaction contacts of the complexes exhibit a constant behavior throughout the 120 ns simulation time.

The RMSF trajectory data revealed that the fluctuations among most amino acids ranged from 0.4 to 3.2 Å (see Figure S6). Notably, the initial residues displayed the most substantial variations, spanning from residue 39 to 100. These residues correspond to protein regions serving as loops, which grant greater flexibility of movement. In contrast, amino acids located within the catalytic region displayed lower flexibility. Consequently, their capacity to bind to the target proteins may be linked to the observed stability of interactions.

## 4. Discussion

This study presents the initial in silico predictions of the protein–protein interactions of the putative protein Ermp1 in *S. pombe*. Based on the results, it can be proposed that Ermp1 could play a role in maintaining the homeostasis of Ca^2+^, amino acids, and proteins in the endoplasmic reticulum, an organelle with high protein synthesis and degradation activity. The analysis of the M28 domain of Ermp1 was interesting because two phosphorylation sites near the active center were predicted. Therefore, it is hypothesized that the proteolytic activity of Ermp1 could depend on serine phosphorylation. The molecular docking suggests that Ermp1 could interact preferentially with consensus sequences located in the loops of the proteins Amk2, Ypt5 and Pex12 from the PPI predictions. These interactions suggest that Ermp1 could act as an aminopeptidase, particularly near the N-amino of the Leu and Met residues.

Aminopeptidases have been found to be present in intracellular organelles, cytoplasm, and cell membranes [104]. Their role in protein degradation is crucial, as they cleave peptides generated by the proteasome or hydrolyze free amino acids for recycling and new protein synthesis [105]. It is known that the proteasome releases peptides of 6–24 amino acids, which can be rapidly degraded by cytosolic peptidases [104,105].

For many years, aminopeptidases have been extensively studied in relation to various cellular processes and their implications in the development of different pathologies, such as inflammatory processes, diabetes, and cancer [106]. It has been observed that in cancer cells, the overexpression of aminopeptidases facilitates the exogenous supply of amino acids, which is necessary for their survival and proliferation [104,107]. In addition, it has been described that the tumor microenvironment increases the rate of protein synthesis, which in turn favors the increase in the expression of aminopeptidases [105,108,109].

In vitro studies have suggested that human ERMP1 increases the proliferation and invasion of cancer cells through the PIK3/AKT/mTOR/β-catenin pathway. However, the molecular mechanism is still unclear [4,5,6,7]. This study proposes a possible interaction of Ermp1 with Amk2 and Gsk3 in *S. pombe*. These two signaling proteins regulate the metabolic and transcriptional activity of cells through TOR-dependent processes in response to environmental signals [65,110,111,112,113]. In humans, orthologous proteins of Amk2 and Gsk3 (PRKAB1 and GSK3B, respectively) have been linked to the ER stress response through the modulation of the UPR pathway [114,115,116]. Furthermore, it would be interesting to explore whether there is a correlation between Ermp1 and both Amk2 and Gsk3 in *S. pombe* within signaling pathways such as TOR and UPR, which are highly conserved in mammals, including humans [117,118]. In humans, these pathways are modified in some types of cancer due to ERMP1 overexpression, but the mechanism is unknown. Therefore, study of the Ermp1 in *S. pombe* could help elucidate its function in human cells.

It is important to mention that the selection of appropriate 3D models is essential for molecular-docking analysis. However, currently it has not yet been possible to experimentally determine the three-dimensional structures of a large number of proteins from different organisms of biological interest. For this reason, the availability of suitable templates for homology modeling is usually limited. In this study, the Phyre2 and AlphaFold Protein Structure databases were employed to generate 3D models of the proteins of interest. Both tools are open access, facilitating exploration of bioinformatics analysis of different proteins with satisfactory results [119,120,121]. Nevertheless, the use of other, more powerful modeling tools should be considered for the validation of structural predictions.

Finally, ClusPro 2.0 and BioLuminate 4.5 are molecular-docking tools that have been widely utilized in diverse biological systems due to their automated computational system and the quality of the predictions, which exceed 50% of the success rate compared to other tools [122,123,124,125]. Since the predictions in both programs were similar, it can be suggested that they are congruent; however, other tools could be used to confirm the results.

## 5. Conclusions

This bioinformatics study proposes the first prediction of the PPIs of Ermp1 with 45 proteins in *S. pombe*, which are highly conserved in humans. According to the GO classification, this group of proteins is mostly related to the transport of calcium in the ER and the metabolism of amino acids and proteins. Therefore, it is very possible that Ermp1 is also related to these processes. The results of the molecular docking and dynamics of the M28 domain with Amk2, Ypt5 and Pex12 support the idea that Ermp1 could act as an aminopeptidase with a preference to hydrolyze the N-amino of leucine and methionine residues. It is important to remember that enzymatic assays are necessary to confirm these predictions. In conclusion, these results provide the basis for future experimental research, which will contribute to the elucidation of the cellular function of other Fxna peptidases, such as human Ermp1.

## Figures and Tables

**Table 1:** Prediction of PROSPER cleavage sites of Ermp1 target proteins in *S. pombe*.

Human Protein	UniProt ID	Ortholog SCHPO ^1^	UniProt ID	MMP ^2^ Prediction	Segment	Position	Score ^3^
PRKAB1	Q9Y478	Amk2	P78789	MMP9	RAQS 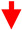 MISI	Met40	1.16
RAB5C	P51148	Ypt5	P36586	MMP9	SLAP 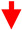 MYYR	Met83	1.08
PEX12	O00623	Pex12	Q8TFH8	MMP9	FWRL 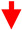 MI	Met342	1.22
CYB5B	O43169	Oca8	Q9USM6	MMP9	GEEV 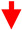 LVDL	Leu45	0.98
FIS1	Q9Y3D6	Fis1	Q9USZ8	MMP9	EALK 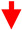 LKNR	Leu117	1.05
ATP2B2	Q01814	Pmc1	Q9HDW7	MMP9	TTMA 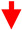 MRTE	Met429	1.23

^1^ *Schizosaccharomyces pombe*. ^2^ Metalloproteinase. ^3^ Values greater than 0.8 increase the confidence of predicting proteolytic cleavage. 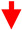 Cleavage site.

**Table 2:** Homology modeling in Phyre2 of Ermp1 target proteins in *S. pombe*. Model validation performed using PDBeFold and PROCHECK-SAVES v6.0.

Protein	Template	Organism	PDB ID	Identity Percentage	Q-Score ^1^	RMSD ^2^ (Å)	Ramachandran Plot ^3^
Ypt5	Rab11	*H. sapiens*	2D7C [90]	43%	0.95	0.33	87.1%
Pex12	Ring 3 ligase	*S. cerevisiae*	4R7E [91]	28%	0.71	0.71	85.1%
Oca8	Cytochrome b5	*B. taurus*	1M2I [92]	50%	1.00	0.00	90.3%
Fis1	Fis1	*H. sapiens*	1NZN [93]	25%	0.90	0.49	90.0%
Pmc1	SERCA2b	*H. sapiens*	6LLE [94]	28%	0.80	0.47	87.5%

^1^ Q-score: quality of alignment, the highest score = 1. ^2^ RMSD: the lower the RMSD, the better the model is in comparison to the target structure. ^3^ Ramachandran plot: a good quality model would be expected to find more than 85–90% in the most favored regions.

**Table 3:** Prediction of cleavage sites by docking of target proteins and Ermp1 in *S. pombe*. Comparison of molecular-docking results in ClusPro and BioLuminate.

Ligand SCHPO ^1^	Docking Prediction	Docking Program	PIPER Energy Score	Cluster Size
Amk2	RAQS 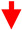 MISI	ClusPro BioLuminate	−923.2 −951.3	28 57
Ypt5	S 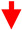 LAPMYYR	ClusPro BioLuminate	−645.5 −638.4	31 28
Pex12	FWR 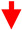 LMI	ClusPro BioLuminate	−680.8 −667.4	124 78
Oca8	GEEVLVD 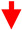 L	ClusPro BioLuminate	−500.6 −542.6	62 54
Fis1	EALKLKN 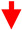 R	ClusPro BioLuminate	−607.1 −633.0	92 74
Pmc1	TT 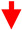 MAMRTE	ClusPro BioLuminate	−1137.7 −1212.5	35 20

^1^ *Schizosaccharomyces pombe*. 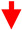 Cleavage site.

## Data Availability

The authors confirm that the data supporting the findings of this study are available within the article.

## Supplementary Materials

The following supporting information can be downloaded at https://www.mdpi.com/article/10.3390/cimb46050280/s1, Figure S1: Three-dimensional structures of target proteins of Ermp1 from *S. pombe*. Homology modeling in Phyre2 and Ramachandran plot. Model validation performed using the PROCHECK-SAVES v6.0 server. Editing of the model performed in UCSF Chimera 1.17.1; Figure S2: AlphaFold prediction and Ramachandran plot of the 3D structure of Amk2 from *S. pombe*; Figure S3: Molecular docking of the M28 domain of Ermp1 with protein targets. Ligand cleavage segment is shown in blue. Cleavage residues are shown in pink. Solvation performed in the WaterMap tool. Editing of the models performed in Maestro 13.0; Figure S4: DIMPLOT 2D-interaction plot of the M28 domain of Ermp1 with protein targets (A–F). The horizontal dashed line represents the interface. The green dotted line represents the hydrogen bond length, while the arc represents the hydrophobic interaction. The dotted boxes delineate the identified interactions between the residues of Ermp1’s catalytic cavity and the potential cleavage residues within the target proteins; Figure S5: Representation of the solvation of the interaction complexes of the M28 domain of Ermp1 and cleavage sites of protein targets. Binding sites of Zn^2+^ are shown in orange. Catalytic site is shown in purple. Catalytic stabilizer is shown in magenta. Cleavage residues are shown in pink. Solvation performed in the WaterMap tool. Editing of the models performed in Maestro 13.0; Figure S6: Analysis of the RMSF trajectories of residues of the protein–protein complexes. The root mean square fluctuation (RMSF) was calculated to examine the residual fluctuations throughout the simulation time for Ermp1–Amk2, Ermp1–Pex12, and Ermp1–Ypt5 (A–C, respectively). Each simulation was conducted for 120 ns in the Desmond Molecular Dynamics System to assess the stability of the protein–protein interaction; Table S1: Comparison of orthologous proteins that interact with ERMP1/Ermp1 in humans and *S. pombe*. GO cellular component, biological process annotation, DIOPT score, similarity, and identity percentages. Proteins highlighted in gray were selected for proteolytic propensity analysis and molecular docking; Table S2: Predictions of the phosphorylation sites in NetPhos 3.1 of human ERMP1; Table S3: Predictions of the phosphorylation sites in NetPhos 3.1 of Ermp1 from *S. pombe*.
