# Supplementary material for: In Silico Analysis of Protein–Protein Interactions of Putative Endoplasmic Reticulum Metallopeptidase 1 in Schizosaccharomyces pombe
Source: Curr Issues Mol Biol. 2024 May 12;46(5):4609–29. doi: 10.3390/cimb46050280 (PMC11120530; doi:10.3390/cimb46050280)

Supplementary material

Figure S1. 3D structures of target proteins of Ermp1 from *S. pombe*. Homology modeling in Phyre2 and Ramachandran plot. Model validation by using PROCHECK-SAVES v6.0 server. Editing the model in UCSF Chimera 1.17.1.

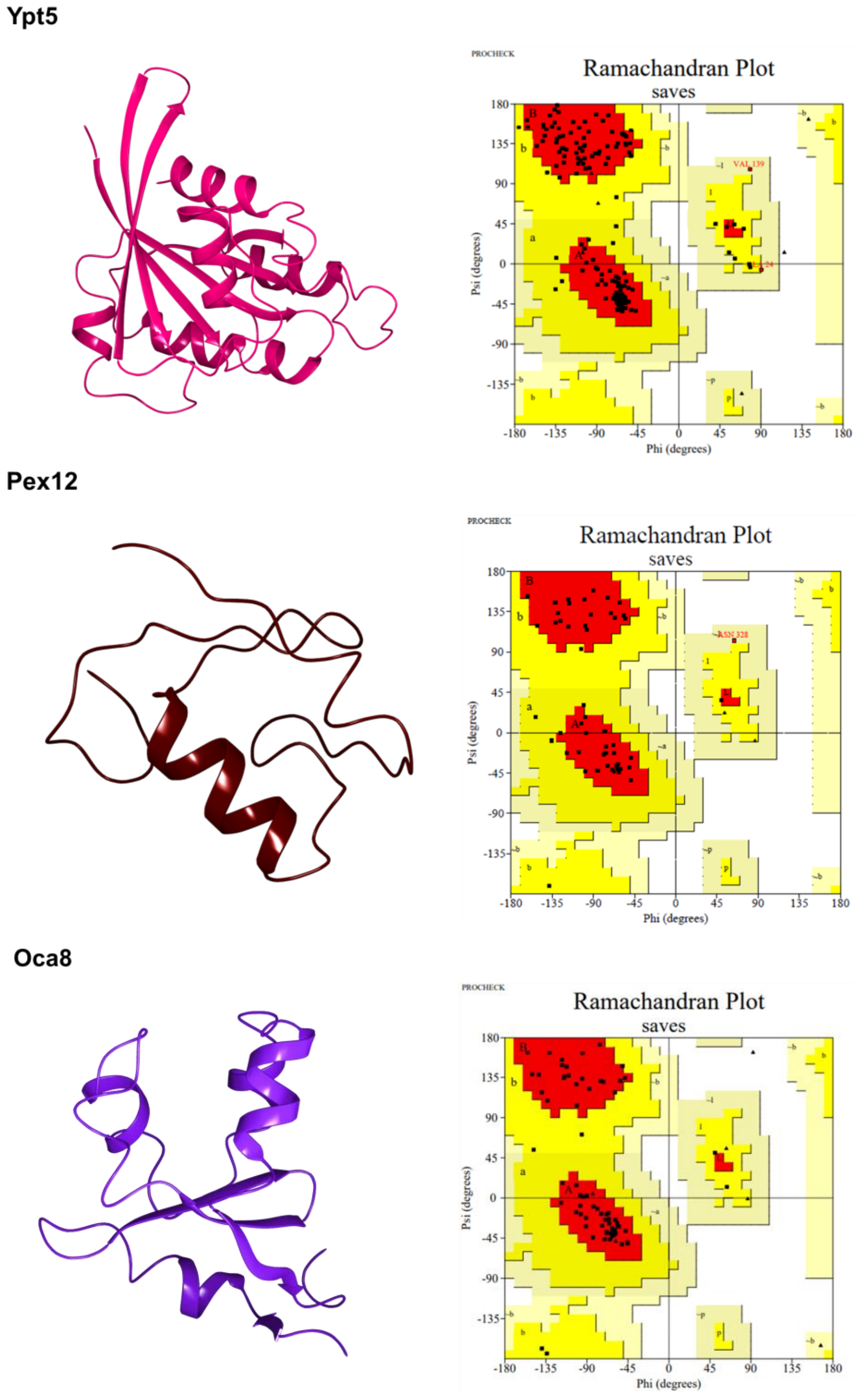

Fis1

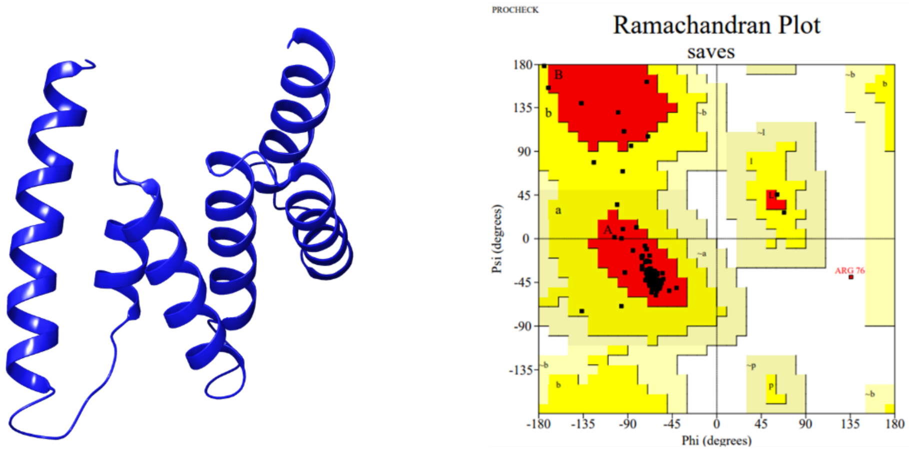
PROCHECK
Ramachandran Plot
saves
Psi (degrees)
Phi (degrees)
ARG 76

Pmc1

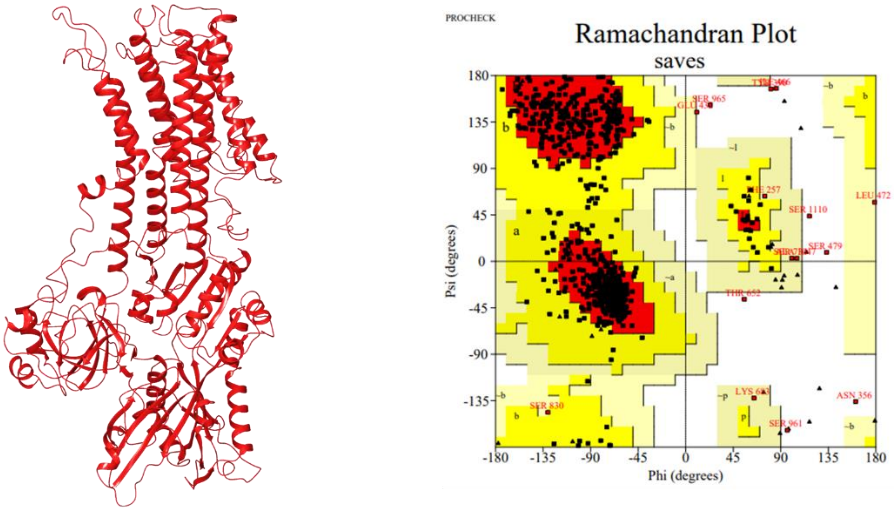
PROCHECK
Ramachandran Plot
saves
Psi (degrees)
Phi (degrees)
SER 965
SER 1110
LEU 472
SER 479
THR 652
ASN 356
SER 830
SER 961

Figure S2. AlphaFold prediction and Ramachandran Plot of the 3D structure of Amk2 from *S. pombe*.

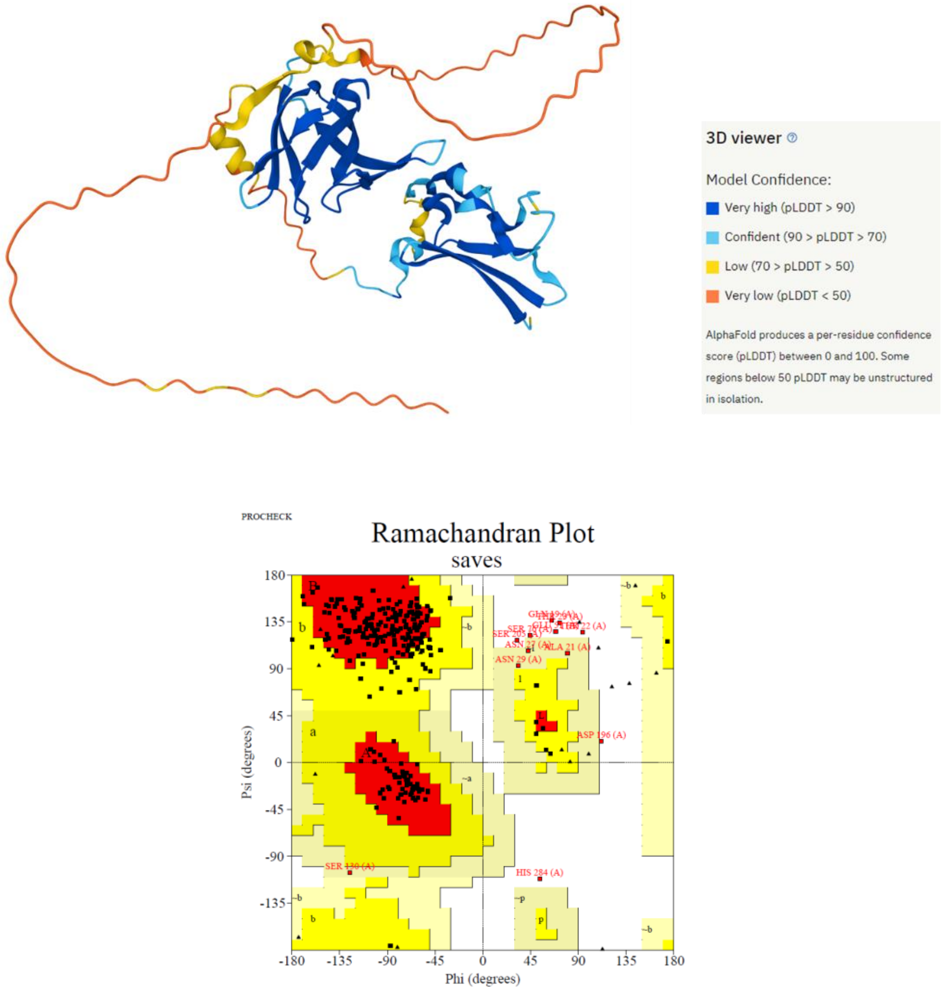

Figure S3. Molecular docking of the M28 domain of Ermp1 with protein targets. Ligand cleavage segment is shown in blue. Cleavage residues are shown in pink. Solvation performed in the WaterMap tool. Editing the models in Maestro 13.0.

Ermp1-Amk2

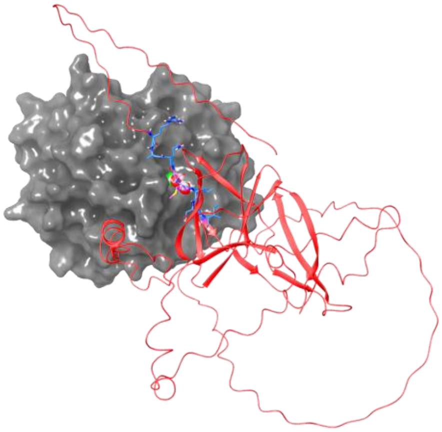

Ermp1-Ypt5

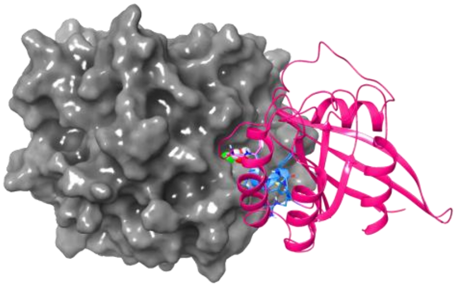

Ermp1-Pex12

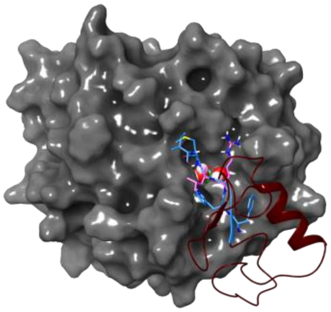

Ermp1-Oca8

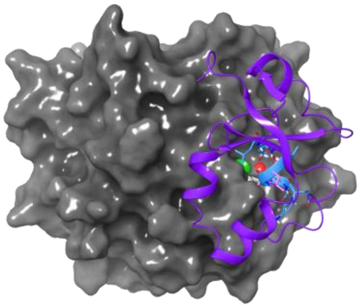

Ermp1-Fis1

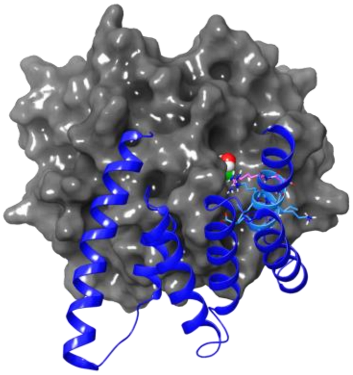

Ermp1-Pmc1

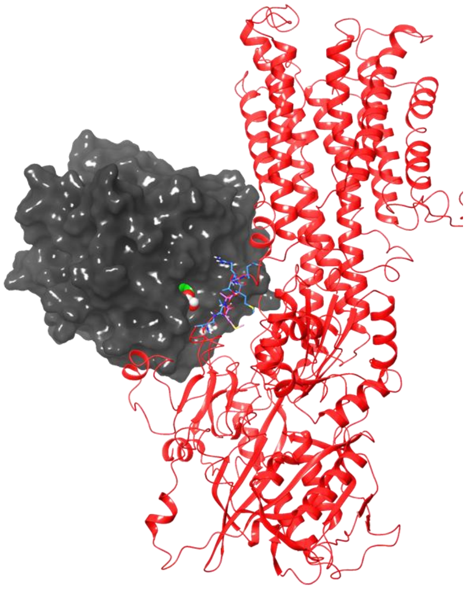

Figure S4. DIMPLOT 2D-interaction plot of M28 domain of Ermp1 with protein targets (A-F). The horizontal dashed line represents the interface. The green dotted line represents the hydrogen bond length, while the arc represents the hydrophobic interaction. The dotted boxes delineate the identified interactions between the residues of Ermp1's catalytic cavity and the potential cleavage residues within the target proteins.

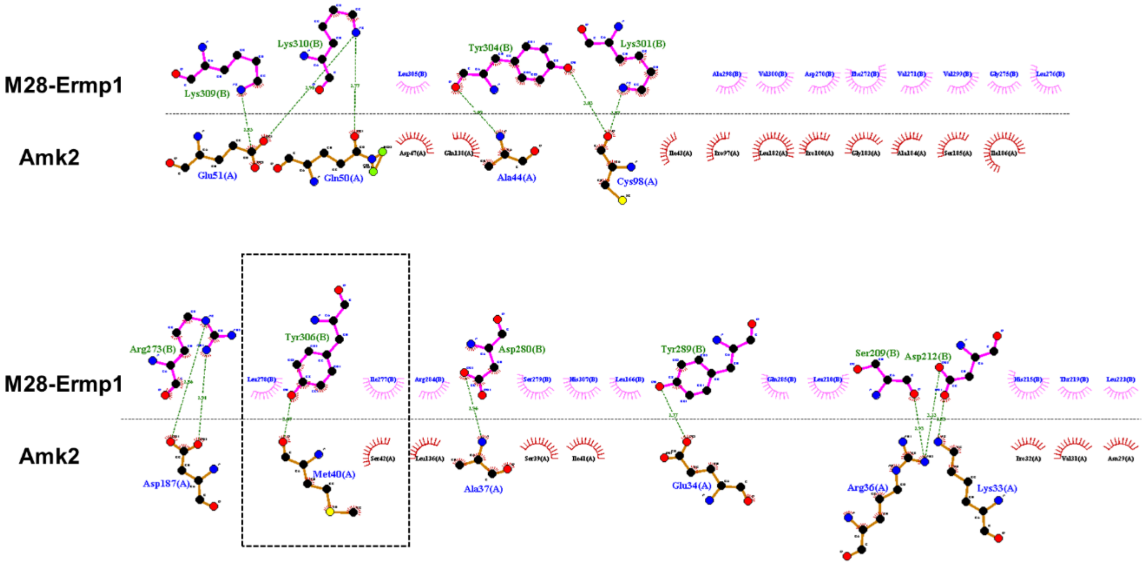

A) Ermp1-Amk2

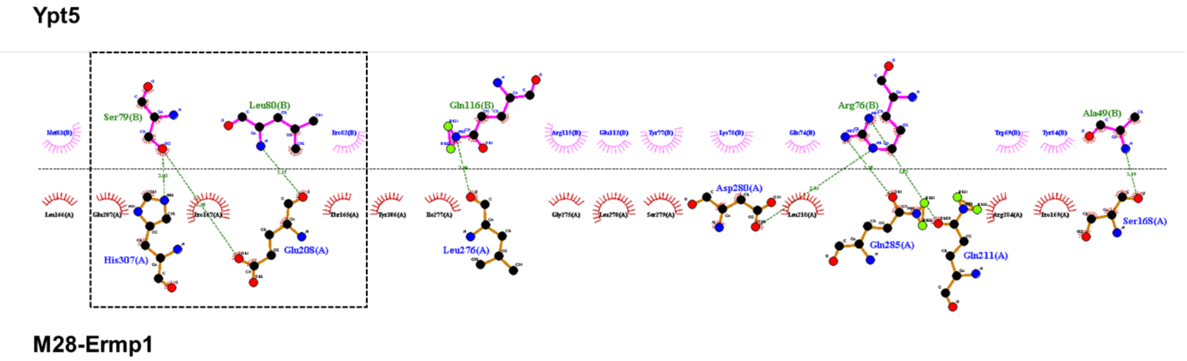

B) Ermp1-Ypt5

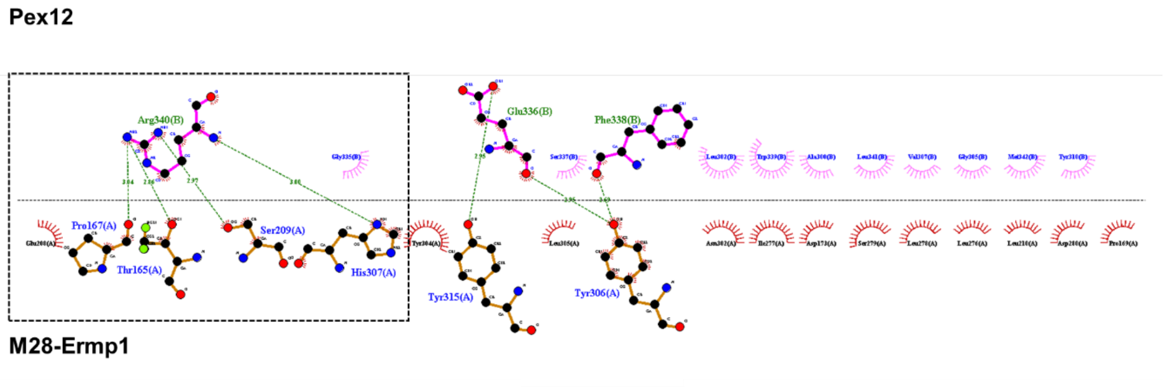

C) Ermp1-Pex12

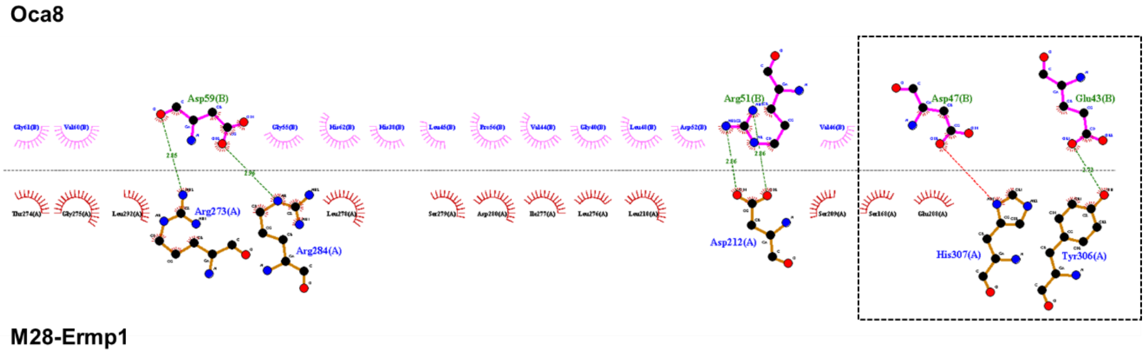

D) Ermp1-Oca8

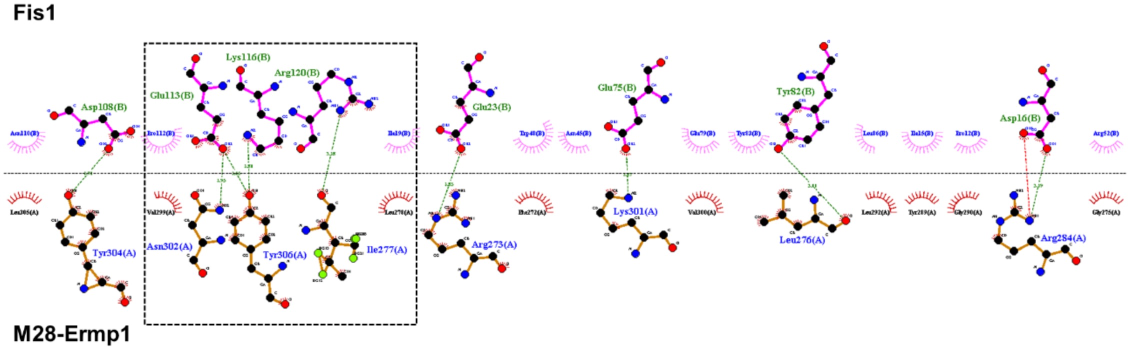

E) Ermp1-Fis1

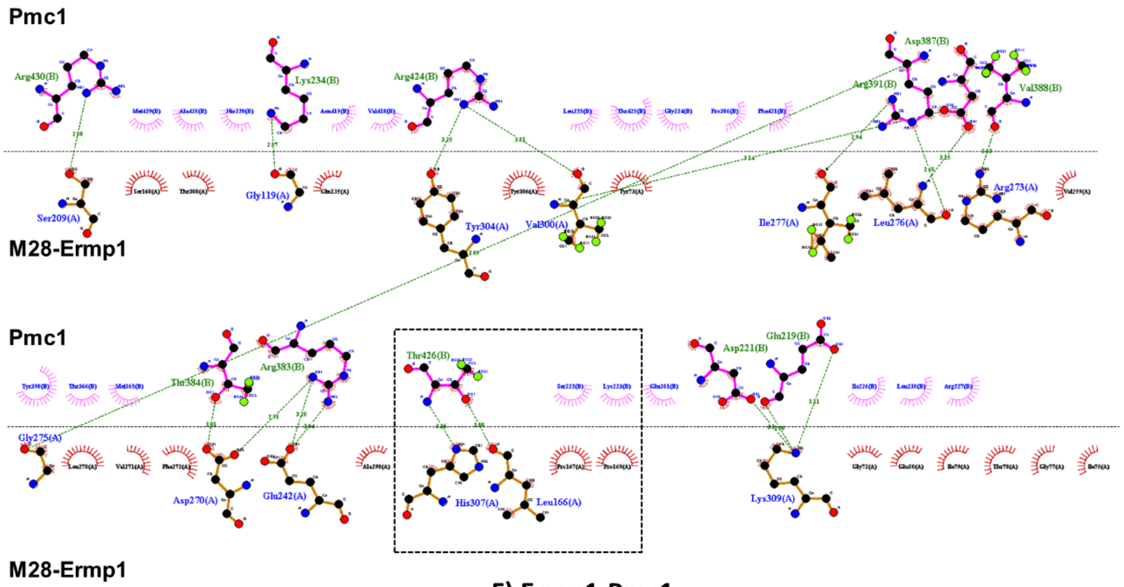
Pmc1
Arg430(B)
Lys234(B)
Arg424(B)
Asp387(B)
Arg391(B)
Val388(B)
Ser209(A)
Gly119(A)
Tyr304(A)
Val300(A)
Ile277(A)
Leu276(A)
Arg273(A)
M28-Ermp1
Pmc1
Thr384(B)
Arg383(B)
Thr426(B)
Asp221(B)
Glu219(B)
Gly275(A)
Asp270(A)
Glu242(A)
His307(A)
Leu166(A)
Lys309(A)
M28-Ermp1

F) Ermp1-Pmc1

Figure S5. Representation of the solvation of the interaction complexes of the M28 domain of Ermp1 and cleavage sites of protein targets (A-F). Binding sites Zn^{2+} are shown in orange. Catalytic site is shown in purple. Catalytic stabilizer is shown in magenta. Cleavage residues are shown in pink. Solvation performed in the WaterMap tool. Editing the models in Maestro 13.0.

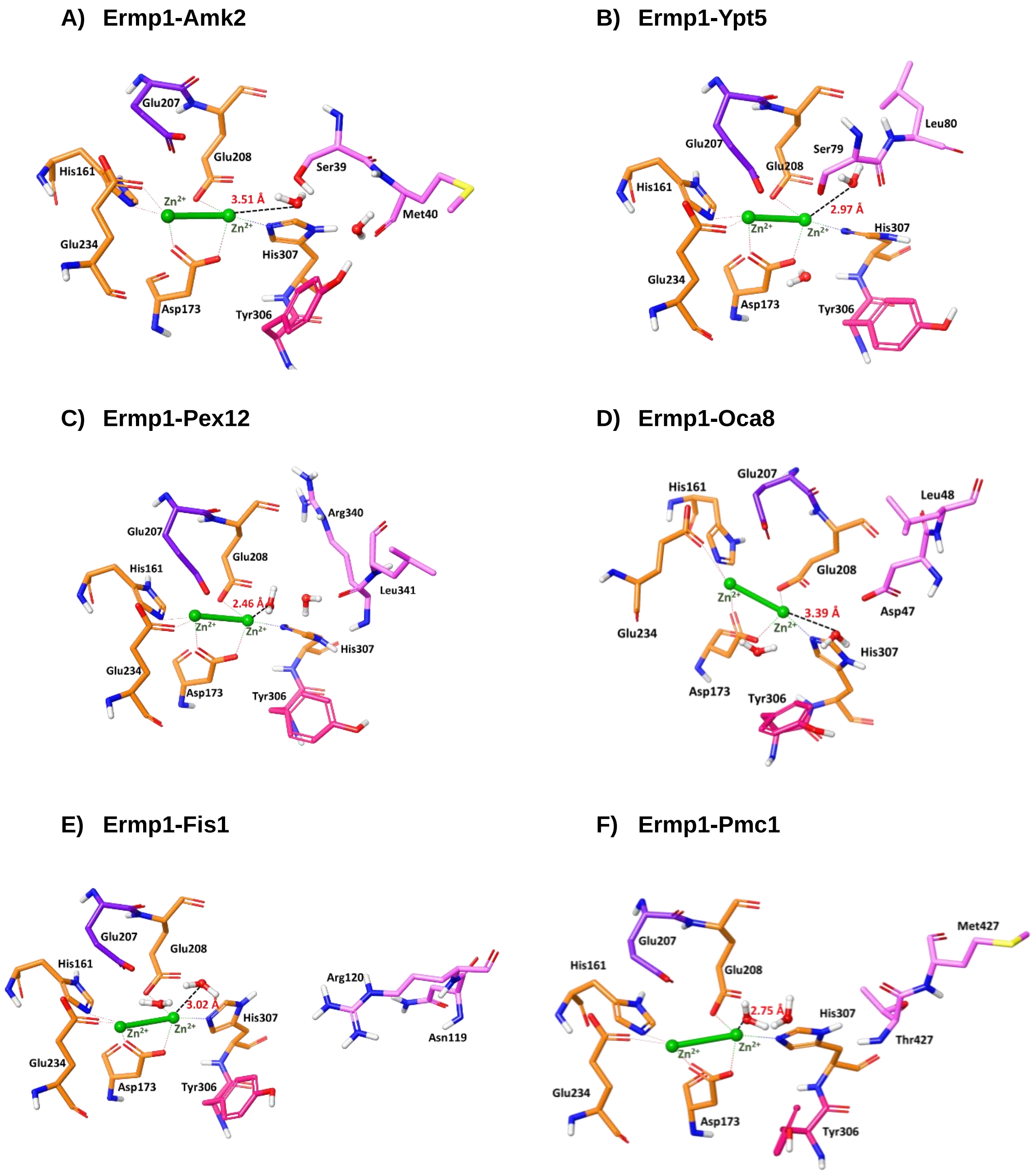

Figure S6. Analysis of RMSF trajectories of residues of the protein-protein complexes. The Root Mean Square Fluctuation (RMSF) was calculated to examine residual fluctuations throughout the simulation time for Ermp1-Amk2, Ermp1-Pex12, and Ermp1-Ypt5 (A-C respectively). Each simulation was con-ducted for 120 ns in the Desmond Molecular Dynamics System to assess the stability of protein-protein interaction.

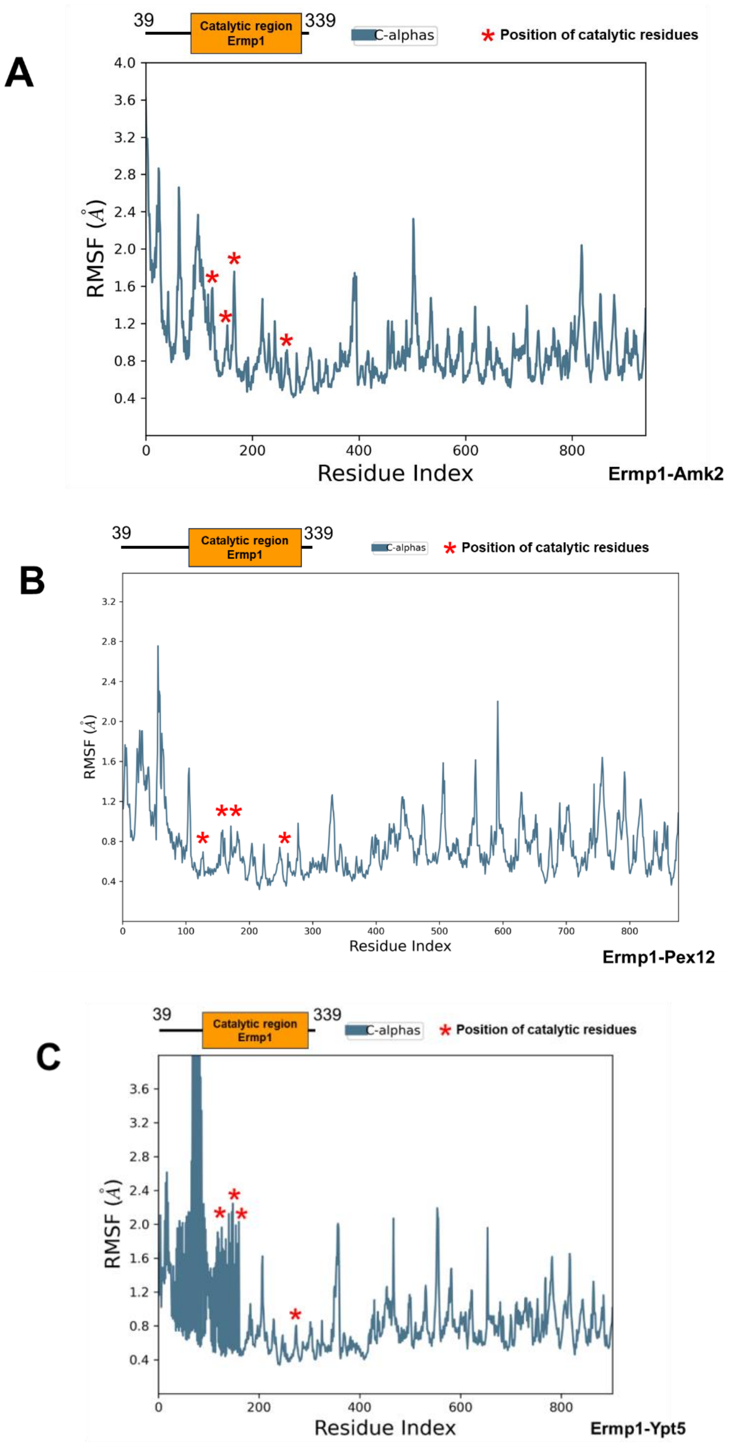

Supplement: Supplementary file 1 [file cimb-46-00280-s001.zip › Supplementary material Figures S1-S6_cimb-2916902.pdf]
