# Supplementary material for: In Silico Analysis of Protein–Protein Interactions of Putative Endoplasmic Reticulum Metallopeptidase 1 in Schizosaccharomyces pombe
Source: Curr Issues Mol Biol. 2024 May 12;46(5):4609–29. doi: 10.3390/cimb46050280 (PMC11120530; doi:10.3390/cimb46050280)
Supplement: Supplementary file 1 [file cimb-46-00280-s001.zip › Supplementary material Table S1_cimb-2916902.pdf]

Table S1. Comparison of orthologous proteins that interact with ERMP1/Ermp1 in human and *S. pombe*. Go cellular component, biological process annotation, DIOPT score, similarity, and identity percentages. Proteins highlighted in gray were selected for proteolytic propensity analysis and molecular docking.

Human protein	GO cellular component	GO biological process	Orthologs Fission Yeast	GO cellular component	GO biological process	DIOPT Score	Similarity percentage	Identity percentage
PGRMC1	ER, mitochondria	Heme biosynthetic process	SPAC25B8.01 (Dap1)	ER	Lipid metabolic process	14	61	44
ERCC4	Nucleus	DNA repair	SPCC970.01 (Rad16)	Nucleus	DNA repair	13	54	32
PRKAB1	Cytosol, nucleus	Signal transduction	SPCC1919.03c (Amk2)	Cytosol, nucleus	Signaling, cell adhesion, conjugation with cellular fusion	13	55	38
EMC2	ER	Protein insertion into ER membrane	SPBC15C4.01c (Oca3)	ER	Protein insertion into ER membrane	12	47	28
ATP6V0C	Plasma membrane	Proton transmembrane transport, endosomal lumen acidification	SPAC1B3.14 (Vma3)	ER, vacuole	Transmembrane transport, proton	12	89	76
GSK3B	Cytosol, nucleus	Signal transduction	SPAC1687.15 (Gsk3)	Cytosol, nucleus	Signaling	12	40	22
EMC1	ER	Protein insertion into ER membrane	SPAC25H1.07 (Emc1)	ER	Protein insertion into ER membrane	12	54	34
TM9SF4	Golgi, endosomes	Protein localization to membrane	SPBC1105.08 (Emp70)	Golgi, endosomes	Protein localization to membrane	12	47	36
TMEM30B	Plasma membrane	Lipid transport	SPBC11B10.07c (Ivn1)	ER	Membrane organization, phospholipid-translocating ATPase complex	12	76	62
CYB5B	ER, mitochondria	Electron transport	SPCC16A11.10c (Oca8)	ER	Lipid metabolic process, electron transfer activity	12	61	38
PEX12	Peroxisome	Peroxisome organization, ubiquitin-protein transferase activity	SPAPB17E12.03 (Pex12)	Peroxisome	Peroxisome organization, ubiquitin-protein transferase activity	11	40	21
RAB5C	Endosomes	Intracellular protein transport	SPAC6F6.15 (Ypt5)	Cytosol, endosomes	Intracellular protein transport	11	78	68
ATP2B2	Plasma membrane	Calcium ion transport	SPAPB2B4.04c (Pmc1)	Vacuole	Calcium ion transport	11	49	34

IER3IP1	ER	Endoplasmic reticulum to Golgi vesicle-mediated transport	SPAC19A8.09 (Yos1)	ER, Golgi	Endoplasmic reticulum to Golgi vesicle-mediated transport	10	67	45
WDR5	Nucleus	Transcription regulation, histone binding	SPBC354.03 (Swd3)	Nucleus, cytosol	Transcription regulation, histone binding	10	45	27
BSCL2	ER	Lipid droplet formation, Lipid metabolism	SPAC3A11.04 (Sei1)	ER	Lipid droplet formation, Lipid metabolism	10	34	24
NCEH1	Plasma membrane, ER	Lipid catabolic process	SPAC1039.03	Cytosol, nucleus	Lipid catabolic process	10	58	38
FIS1	Mitochondria	Mitochondrial membrane fission	SPBC11G11.01 (Fis1)	Mitochondria	Mitochondrial membrane fission	10	62	29
MPPE1	Golgi, ER	ER-Golgi transport, GPI anchor biosynthetic process	SPAC630.12	ER	GPI anchor biosynthetic process	9	40	23
CCDC47	ER	Protein insertion into ER membrane, chaperone	SPBC2G5.01	ER	Protein insertion into ER membrane, chaperone	9	41	22
SLC23A2	Plasma membrane	Transmembrane transport	SPAC1399.01c	Vacuole, Plasma membrane	Transmembrane transport nucleobases	8	39	21
SLC13A4	Plasma membrane	Transmembrane transport	SPBC3B8.04c (Plt1)	ER, plasma membrane	Transmembrane transport, phosphate	8	37	21
SLC31A2	Plasma membrane	Transmembrane transport	SPBC23G7.16 (Ctr6)	Vacuole, Plasma membrane	Transmembrane transport, cupper	7	45	25
SPAG4	Nucleus	Cell differentiation	SPBC12D12.01 (Sad1)	Nucleus	Meiotic nuclear division	7	48	28
SLC5A6	Plasma membrane	Transmembrane transport	SPBC23G7.13c	Plasma membrane	Transmembrane transport, urea	5	41	19
SLC7A14	Plasma membrane, lysosomes	Amino acid transport	SPAP7G5.06 (Per1)	Golgi, Plasma membrane	Transmembrane transport, amino acid	5	36	21
ATP2A1	ER	Calcium ion transmembrane transport	SPBC31E1.02c (Pmr1)	ER, Golgi, plasma membrane	Calcium ion transmembrane transport	5	51	35

NUP35	Nucleus	Nucleocytoplasmic transport	SPAC19E9.01c (Nup40)	Nucleus	Nucleocytoplasmic transport, rRNA export from nucleus	5	38	24
TRHDE	Plasma membrane	Peptide catabolic process	SPBC1921.05 (ape2)	Vacuole, cytosol	Peptide catabolic process	5	49	28
ATP2A3	ER	Calcium ion transmembrane transport	SPBC31E1.02c (Pmr1)	ER, Golgi, plasma membrane	Calcium ion transmembrane transport	5	51	34
YWHAH	Cytosol	Signal transduction	SPAC8E11.02c (Rad24)	Cytosol, nucleus	Signaling mitosis	4	78	62
SCN4A	Plasma membrane	Sodium ion transmembrane transport	SPAC6F6.01 (Cch1)	Plasma membrane	Calcium ion import across plasma membrane	4	38	21
ENTPD2	Plasma membrane, ER	Nucleoside diphosphate catabolic process	SPCC11E10.05c (Ynd1)	ER, Golgi, plasma membrane	Nucleobase-containing small molecule metabolic process	4	40	26
RNF185	ER, mitochondria	Ubiquitin-dependent protein catabolic process	SPBC17A3.10 (Pas4)	Peroxisome, ER	Ubiquitin-like protein transferase activity	4	48	31
ACPT	Plasma membrane	Acid phosphatase activity	SPBC4.06	Mitochondria	Lipid metabolic process, phospholipid metabolic process	4	33	23
GGT7	Plasma membrane	Glutathione biosynthesis	SPAC664.09 (Ggt1)	Vacuole, ER	Glutathione catabolic process	3	46	27
AQP2	Golgi, Plasma membrane	Water transport	SPAC977.17	Plasma membrane	Water transport	3	42	30
RHCG	Plasma membrane	Ammonia transport	SPCPB1C11.01 (Amt1)	Golgi, plasma membrane	Ammonium transmembrane transport	3	37	22
SLC39A5	Plasma membrane	Zinc ion transmembrane transport	SPAP8A3.03 (Zip3)	ER	Zinc ion transmembrane transport	2	48	28
ELAVL1	Nucleus	3'-UTR-mediated mRNA stabilization	SPAC343.07 (Mug28)	Nucleus	RNA binding	2	39	22
ZDHHC12	ER, Golgi	Protein palmitoylation	SPBC3H7.09 (Erf2)	ER, Golgi	Protein palmitoylation	2	41	26
AQP6	Plasma membrane	Water transport	SPAC977.17	Plasma membrane	Water transport	1	42	27

ISG15	Cytosol	Ubiquitin protein ligase binding	SPAC1805.12c (Ubi2)	Cytosol, nucleus	Ubiquitin protein ligase binding	1	57	37
CREB3L1	Nucleus, ER	Regulation of transcription by RNA polymerase II	SPBC29B5.01 (Atf1)	Nucleus	Regulation of DNA-templated transcription	1	32	20
RXFP1	Plasma membrane	Hormone-mediated signaling pathway, extracellular matrix organization	SPBC887.09c (Sog2)	Cytosol, nucleus	Signaling, establishment or maintenance of cell polarity	1	40	26
SLC22A23	Plasma membrane	Transmembrane transporter activity	SPAC20G8.03 (Itr2)	Plasma membrane, ER	Transmembrane transport, inositol	1	38	21
